# Land Carrying Capacity in China: A Perspective on Food Nutritional Demand

**DOI:** 10.3390/foods12244378

**Published:** 2023-12-05

**Authors:** Jinyi Zhang, Li Tan, Dong Ai, Fei Lun, Nan Wang, Mengbing Wu, Jinmin Hao

**Affiliations:** 1 College of Land Science and Technology, China Agricultural University, Beijing 100193, China; 18638366770@163.com (J.Z.); aidong@cau.edu.cn (D.A.); lunfei@cau.edu.cn (F.L.); 2 Key Laboratory of Agricultural Land Quality, Ministry of Natural Resources, Beijing 100193, China; 3 Land Consolidation and Rehabilitation Center, Ministry of Natural Resources, Beijing 100035, China; tanli@lcrc.org.cn (L.T.); wangnan_1994@126.com (N.W.); 4 Key Laboratory of Land Consolidation and Rehabilitation, Ministry of Natural Resources, Beijing 100035, China; 5 Faculty of Architecture, Building and Planning, The University of Melbourne, Victoria 3010, Australia; iceywu1128@gmail.com

**Keywords:** land carrying capacity, food nutrition, food security, agricultural restructuring, China

## Abstract

The sustainable and stable population support capacity of a country or region is of great concern. This study proposes a new method for evaluating the land carrying capacity (LCC) based on food nutrition demand and establishes a clear link between nutritional health and land. We delved into the evolving dynamics of food consumption and production structures in China between 1990 and 2020, with a focus on the spatial variations among its 31 provinces. The objectives of this study were to assess the status of LCC, identify the critical nutritional factors constraining LCC enhancement, and propose differentiated pathways for improving LCC. The results showed that: (1) There has been a steady increase in the annual consumption of animal-based products, while plant-based product consumption has declined. (2) Overall, food supply capacity has expanded, displaying an “east high, west low” trend, resulting in an imbalanced food supply level. (3) The LCC for energy and carbohydrates exhibited continuous fluctuating growth but displayed a declining trend after 2018. (4) The pressure on land carrying capacity has shifted from a state of “surplus” to “abundant surplus,” signifying a safe food system level. However, significant spatial variations persist, leading to shortages and surpluses. Therefore, this work suggests that addressing these disparities requires the optimization of food consumption structures and increasing the supply of animal-based foods. This approach leverages regional advantages and reduces disparities in regional LCCs. This study provides a valuable reference for ensuring food security in response to unprecedented global changes in sustainable development.

## 1. Introduction

The concept of “Land carrying capacity” (LCC) is used to assess the relationship between human socioeconomic activities and land use [1], referring to the population that a piece of land can sustain. Meanwhile, it also considers the equilibrium between food consumption and food production, human demands, and available resources [2]. The LCC index (LCCI) serves as a metric to gauge the pressure on local land resources. Concerns over LCC gained prominence due to food crises, environmental degradation, and resource shortages since the 1960s and 1980s all over the world [3]. Recent years have seen an additional 130 million people worldwide facing acute and severe food insecurity, primarily due to the economic impact of the COVID-19 pandemic [4]. Moreover, the Russia–Ukraine conflict has caused a further shock, resulting in record-breaking increases in food prices [5,6,7,8]. The question of how many people a country or region can sustainably and steadily support is a common concern in both academic and political circles.

Studies focused on land carrying capacity (LCC) that are based on limiting factors consider that the carrying capacity of an area is determined by resource scarcity and emphasize the determinacy of a single factor in determining LCC. This approach traces back to Allan’s 1949 proposal of a formula for calculating LCC, particularly with respect to grain-based land products [9]. With global socioeconomic development and the improvement of living standards, dietary nutrient structure have evolved, with a growing emphasis on nutrition due to increased income among residents [10]. Numerous studies have demonstrated that producing one unit of livestock products requires over three times more land than producing one unit of grain [11,12]. The rising demand for meat, eggs, and dairy agricultural products leads to an annual increase in land resource demand and external dependence [13,14,15,16], ultimately diminishing the LCC of the region. The viewpoint alights with empirical analyses conducted by Peters et al. in New York (USA) [17], Ferng in Taiwan (China) [18], and Zhao et al. in China [19]. Expanding the LCC concept from single grains to encompass vegetables, fruits, meat, eggs, milk, and aquatic products within a complete dietary system represents a broader and more refined understanding of LCC based on limiting factors. However, research on scientifically evaluating LCC based on food nutrition is notably lacking, despite the diversity of food choices. Therefore, there is an urgent need for a new perspective to scientifically and rationally evaluate the relationship between food and population. This approach will provide a basis for identifying LCC constraints, enhancing the nutritional quality of the population’s diet, and advancing the development of sustainable food systems. 

Over recent decades, agricultural development in China has achieved remarkable results, accompanied by a constant evolution in its food consumption. Notably, grain consumption has been decreasing, whereas other foods have seen significant growth, such as fruits, vegetables, meat, eggs, milk, and aquatic products [20,21,22,23,24]. The challenge of food security is gradually shifting from merely ensuring subsistence to meeting nutritional demands. It is important to note that food, nutrition, and health are inextricably linked. Unhealthy diets account for a significant proportion of chronic diseases in China [25]. In response to these challenges, the 20th CPC National Congress has prioritized “Healthy China” as a fundamental aspect of China’s overall development goal for 2035 and has proposed the strategic importance of safeguarding people’s health and improving policies to promote public well-being [26]. However, traditional food consumption is deeply rooted in historical and cultural traditions, coupled with an emphasis on quantitative food goals; thus, it has profoundly influenced land-use policies and regulations. A policy focus on cereals has limited the ability of agriculture to diversify in response to the burgeoning market demand [27]. China has implemented one of the strictest global cultivated land protection systems, with food security being the fundamental development goal [28]. However, food security has often been equated with staple grain self-sufficiency, overshadowing the need for food diversity and nutritional considerations, resulting in a lag in supply-side restructuring [27]. Although some studies have explored LCC from a food nutrition perspective, there remains a dearth of long-term studies in China, and the global and regional variations are not well understood [29,30]. China now faces the task of broadening its perspective on food security to elucidate the intricate relationship between energy and nutritional supply and demand. A key issue to address is how to use limited land resources to ensure that everyone has access to energy-sufficient and nutritionally diverse food.

To address this gap, this study drew on research findings in the field of nutrition [31], used energy and nutrient values as standardized measures for all food items [32], constructed a food–nutrition conversion model, and established a dietary nutrition-based LCC evaluation methodology. Next, we used this model to comprehensively examine the spatial and temporal characteristics of food nutrient supply and demand in China. Finally, the study determined the population that could be sustained by land resources and also identified key nutritional constraints on improving China’s LCC. The findings provide empirical support for enhancing the LCC, restructuring agriculture, and promoting sustainable food systems.

Our research has three significant contributions. (1) We discussed the new demand for nutritious food consumption and the new requirement for multiple sources of food supply. (2) We assessed how much of a population can be sustained by land resources in China, analyzed the land carrying pressure situation, and identified what the key factors limiting the increase in LCC are. (3) We addressed how various regions in China could enhance their LCC to ensure universal access to energy-rich and nutrient-dense food for all individuals.

## 2. Materials and Methods

### 2.1. Methodology

#### 2.1.1. Analytical Framework

**Figure 1:**
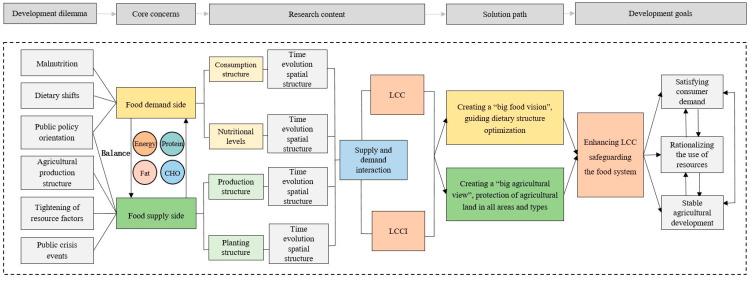
Analytical framework.

To comprehensively study food consumption and supply, as well as their interrelationship, we developed a research framework for LCC based on the lens of food nutrition (Figure 1). The increases in malnutrition, dietary shifts, delayed policy responses, an impractical structure of agricultural production, resource constraints, and the frequent occurrence of public crises have collectively diminished LCC and heightened the vulnerability of food systems. Food security hinges on achieving a delicate equilibrium between supply and demand, and LCC serves as a robust indicator of this equilibrium. LCC essentially assesses the balance between human consumption and food production, as well as the equilibrium between human demand and resource supply. To approach this from a dietary nutrition perspective, this study used the following analytical methods: (1) this study employed the food nutrition conversion method to estimate food consumption, shedding light on the trends and spatial variations in food consumption; (2) it evaluated food supply and analyzed crop cultivation to delve into land use and agricultural production; (3) the LCC and LCCI were assessed based on the real nutritional consumption demands and the constraints imposed by nutritional element shortages; and (4) finally, a theoretical foundation was established for guiding the optimization of dietary nutrient structure on the demand-side demands and adjusting agricultural production to increase on the supply side. These actions aimed to address the developmental needs of various stakeholders and ensure food security.

#### 2.1.2. Food–Nutrition Conversion Model

A total of 12 food categories were included in this study, including 5 crop products (grains, edible oils, vegetables, sugar crops, and fruits) and 7 animal products (pork, beef, mutton, poultry, eggs, milk, and aquatic products) [16,24,29,30,33]. We employed a food–nutrient conversion model to ensure consistent measurements of dietary nutrition. The model calculated the energy and three major nutrients (protein, fat, and carbohydrate), enabling us to examine both the quantity and nutritional quality of food supply and demand. This approach aided in understanding the complete nutritional profile of the diet. In the spatial analysis, we analyzed the spatial variability by visualizing the 2020 food nutrition transformation results in ArcGIS 10.7 through GIS spatial analysis method.

(1) $$NPQ_{j}=\overset{12}{\underset{i=1}{\sum }}PQ_{i}\times EP_{i}\times FLP_{i}\times e_{ij},$$

(2) $$NRQ_{j}=\overset{12}{\underset{i=1}{\sum }}RQ_{i}\times EP_{i}\times FWP_{i}\times e_{ij},$$

where $NPQ_{j}$ is nutrient production, with *j* = 1, 2, 3, and 4 for energy, protein, fat, and carbohydrate, respectively; $NRQ_{j}$ is nutrient consumption;$\;PQ_{i}$ is production of food *i*; $RQ_{i}$ is consumption of food *i*; *EP_i_* is the edible portion of food group *i*, i.e., the percentage of the raw edible portion of a particular food as a percentage of the food [1]; $FLP_{i}$ is the average food loss percentage of the country; $FWP_{i}$ is the average food waste percentage of the country; and $e_{ij}$ is the nutrient content per unit of food.

Food consumption for urban residents before 2013 was in processed cereals and after 2013 is in unprocessed grains, both with a conversion factor of 0.75 [33]. There was a combined oil yield of 40% for oilseed crops [19] and a combined sugar yield of 11.69% for sugar crops [19]. When evaluating food supply, it was essential to take into account the proportion of losses that occur at various stages of food production, including post-harvest handling, storage, processing, and distribution. Similarly, when assessing food demand, it is crucial to consider the proportion of waste that occurs during consumption. These conversion factors are detailed in Table 1 [34,35].

#### 2.1.3. LCC

The LCC is defined as “the maximum population that can be sustained by the productivity of land resources under specific production conditions and living standard” [36]. From the nutritional perspective, the LCC indicates the relationship between a local population and food production. The amount of local land resources in a region that can support the population is determined by converting various types of food into essential nutrients, such as energy, carbohydrates, proteins, and fat [29,30,37], and comparing them with the per capita dietary nutrition requirements of residents. The formula for $LCC_{j}$ is given below.

(3) $$LCC_{j}=\frac{NPQ_{j}}{NRQ_{j}}.$$

In Equation (3), $LCC_{j}$ denotes the land resource carrying capacity (number of individuals) based on energy, proteins, fats, and carbohydrates; $NPQ_{j}$ denotes nutrient production; and $NRQ_{j}$ denotes nutrient consumption. The production and consumption of proteins, fats, and carbohydrates is given in kg, while the production and consumption of energy is given in kcal.

Notably, the LCC based on dietary nutrition seeks an upper limit of the population carrying capacity under specific premises, assuming that there is no material circulation and exchange between regions, and that important influences, such as regional productivity, remain constant. Thus, there is a considerable gap between the real and estimated LCC.

#### 2.1.4. LCCI

The LCCI is an indicator to assess the stress on local land resources calculated by comparing the current population (*P_a_*) to the LCC (Equation (4)). A higher LCCI indicates greater pressure on local land resources and a reduced capacity of local land resources to produce enough food to meet the consumption needs of the population. The regional LCC can be classified into three levels: balance, surplus, and overload. Furthermore, the LCCI can be further subdivided into seven levels based on the degree of surplus or overload (Table 2) [38]. We visualized the representation of these seven levels by means of GIS spatial analysis.

(4) $$LCCI_{j}=\frac{p_{a}}{LCC_{j}}\;$$

### 2.2. Data Resources

Food production data were obtained from the China Statistical Yearbook. Food consumption data were obtained from the China Household Survey Yearbook, China Statistical Yearbook, and China Rural Statistical Yearbook, with fluctuations in 2013 due to changes in the statistical indicators and scope of the National Bureau of Statistics [39]. After 2017, the food consumption statistics of the residents were refined into two parts, urban and rural, and this study was weighted based on the number of people. Population data were obtained from the China Statistical Yearbook. Nutritional composition of food, edible part data from “Chinese Food Composition Table: Standard Edition (6th Edition)”, Vol. 1 and Vol. 2 [40]; China provincial administrative regions vector data were downloaded from the Resource and Environment Science and Data Center, Chinese Academy of Sciences (https://www.resdc.cn/DOI/doi.aspx?DOIid=122 (accessed on 28 March 2022)).

## 3. Results and Analyses

### 3.1. Food Nutritional Demand

**Figure 2:**
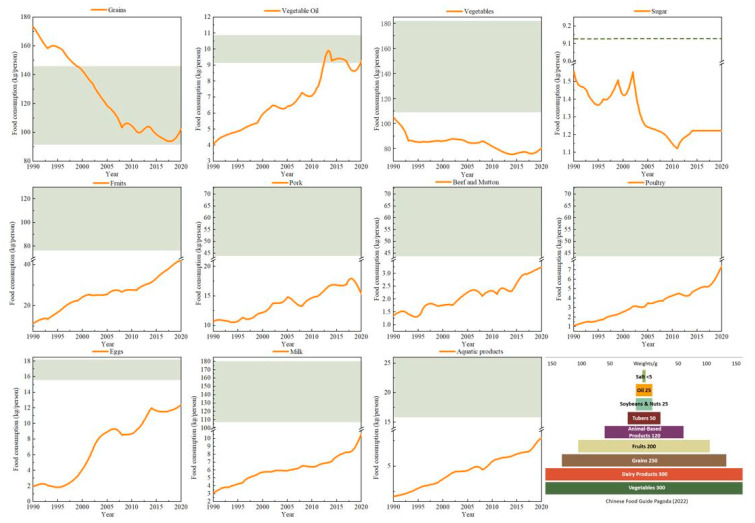
Changing food consumption structure. Note: the green part is the recommended food intake of the Balanced Dietary Pagoda (2022) for Chinese residents [31].

From 1990 to 2020, there was a notable increase in the consumption of land-intensive animal foods, including meat, eggs, and milk, whereas crop food consumption, particularly cereals and vegetables, saw a decline over the years (Figure 2). Despite these shifts, Chinese food consumption was still dominated by cereals, consistent with findings by Yu and Du [24]. Among animal foods, poultry consumption experienced the most substantial increase, marking a 5.85-fold increase with annual per capita consumption rising from 1.08 kg to 7.40 kg. Milk consumption also represents a high increase (5.42-fold), from 1.93 kg per capita to 12.36 kg per capita. However, it is important to note that the share of cereal consumption has been on a declining trend, with an annual per capita reduction of 70.93 kg. Notably, the structure of livestock and poultry meat consumption in China changed considerably between 1990 and 2020. Pork consumption decreased from 81.37% to 59.02% of the total livestock meat consumption, and poultry meat increased from 8.28% to 28.51%. But during the study period, only in 2020 did China’s per capita consumption of animal-based food reach the minimum of the Dietary Guidelines for Residents. This result was consistent with those in Xin’s study [39] on Chinese dietary structure changes. Significant changes in food consumption are caused by many factors, including income growth, urbanization, and market development [10,41,42,43,44]. In the future, animal food consumption will further increase, and the demand for feed will continue to expand based on the increased external dependence on soybeans and corn, which will threaten food security.

**Figure 3:**
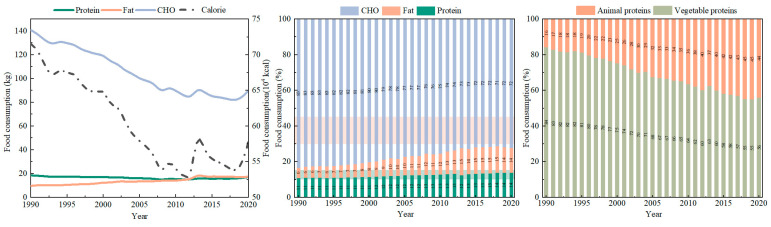
Temporal changes in nutritional structure of food consumption.

The nutritional dietary structure in China was optimized from 1990 to 2020; however, overall carbohydrate consumption far exceeded the recommended values, but fat consumption was below the recommended values, leaving much scope for adjustment. In the past 31 years, carbohydrate consumption declined notably from 140.80 kg/person/year to 89.92 kg/person/year, a decrease of 36.14%. In contrast, fat consumption increased from 9.57 kg/person/year to 17.35 kg/person/year, a substantial increase of 81.18% (Figure 3). This shift is mainly due to a decrease in the consumption of grains and an increase in the consumption of animal foods such as meat. The results were consistent with those of a study conducted by the Chinese Society of Nutrition [31]. In terms of nutrient energy ratios, the ratio of fat–energy increased from 5.68% to 13.95%; however, it is still 6.05% lower than the minimum recommended intake. On the other hand, the ratio of carbohydrate–energy decreased from 83.48% to 72.33%, but it remained 7.33% higher than the maximum recommended ratio. The ratio of protein–energy fell within the recommended range, and it increased to 13.71%. Moreover, the amino acid composition of the protein provided by animal foods is more suited to the needs of the human body, making it a better-quality protein for human needs than the protein provided by plant foods. The structure of protein intake was optimized from 1990 to 2020, with the proportion of animal protein intake increasing by 1.81 times from 15.67% to 43.95%. However, the proportion of vegetable protein in total protein consumption remained higher than that of animal protein consumption.

**Figure 4:**
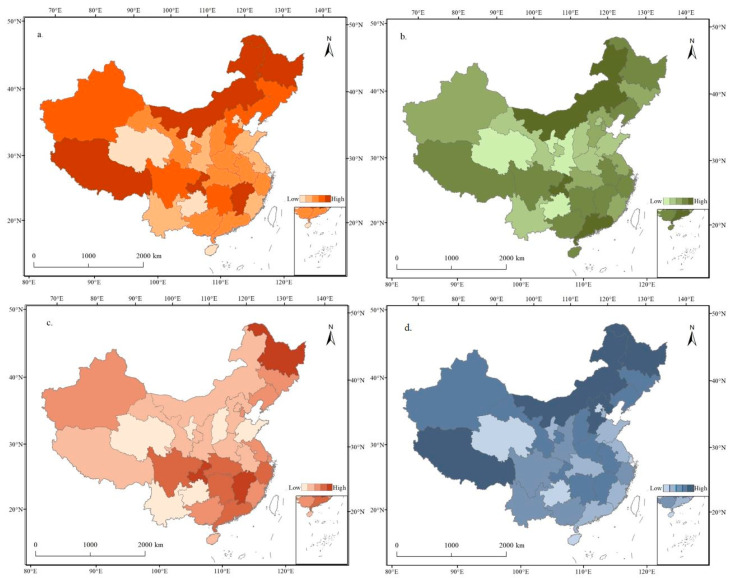
Spatial distribution of food nutrition consumption in 2020. (**a**) Calorie consumption; (**b**) protein consumption; (**c**) fat consumption; (**d**) carbohydrate consumption.

Figure 4 shows that China exhibits significant regional variation in dietary patterns and nutritional consumption. This regional diversity is evident in variations in the intake of macronutrients and energy across different parts of the country. Spatial clustering of the three major nutrients showed distinct patterns. Fat clustering exhibited the highest spatial concentration, followed by protein clustering and carbohydrate clustering. Regions with high fat and protein intake were concentrated in the southern areas of the Yangtze River (Figure 4). Provinces with high energy intake were distributed in bands. In particular, provinces with high energy intake in the northern region included Heilongjiang, Inner Mongolia, Jilin, Liaoning, Xinjiang, and Hebei. In contrast, high energy intake provinces in the southern region included Jiangxi, Hunan, Chongqing, Sichuan, and Tibet. Areas with high protein consumption showed a dispersed multipoint distribution. The top three provinces with high protein intake were Guangdong, Inner Mongolia, and Chongqing, which were the centers, with annual per capita consumptions ranging from 19.02 kg to 20.67 kg. High-fat consumption areas were primarily located in the regions south of the Yangtze River. Carbohydrate consumption patterns tended to align with energy consumption, with Tibet, Inner Mongolia, Heilongjiang, and Hebei exhibiting the highest carbohydrate intake. Provinces such as Zhejiang, Shanghai, Guangdong, and Jiangsu were notable for their high nutritional levels, characterized by a diverse and balanced diet. These regions are considered representative of a healthy Chinese dietary pattern, marked by a variety of foods, mild flavors, lower salt content, and a high intake of vegetables, fruits, fish, prawns, dairy products, and legumes. Such dietary patterns contribute to avoiding nutritional deficiencies and diet-related chronic diseases, resulting in relatively high life expectancies [45]. In contrast, less developed regions, such as Qinghai, Gansu, and Guizhou, had lower nutritional intake across all three nutrient groups. In some areas, nutritional intake was imbalanced, as seen in Henan, Shandong, and Shanxi, where carbohydrates provided a significant proportion of energy, while protein and fat intake fell short of recommended levels (Figure 4).

### 3.2. Food Nutritional Supply

**Figure 5:**
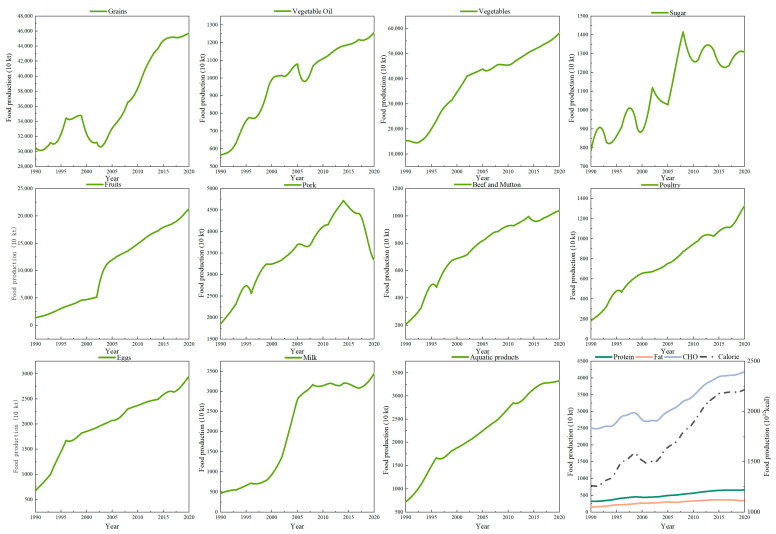
Changing structure of food production.

Between 1990 and 2020, China saw significant increases in the production of major agricultural products (Figure 5), driven by institutional innovation, technological progress, market reforms, and increased agricultural inputs [46]. This growth resulted in an overall increase in food supply capacity. However, the growth rates varied among different food supplies. Fruit, milk, and poultry meat production experienced high growth rates, while pork, sugar, and grain production had relatively low growth rates. Among them, the growth rate of fruits was an impressive 14.31 times higher, while grain production only increased by a factor of 0.5. Notably, the pig industry has faced constraints imposed by local governments because of pollution, epidemics, and other risks since 2015, particularly after the outbreak of African swine fever. This led to a steep decline in pork supplies [47]. Regarding food nutrients and energy supply, we observed a significant increase in quantity with a relatively lower growth rate, averaging 1.77% annually. This is mainly due to the large carbohydrate supply base. On the other hand, the fat supply consistently remained low but had the highest average annual growth rate of 2.80%. The trend in protein supply closely followed that of fat supply, with an average annual growth rate of 2.46%. 

**Figure 6:**
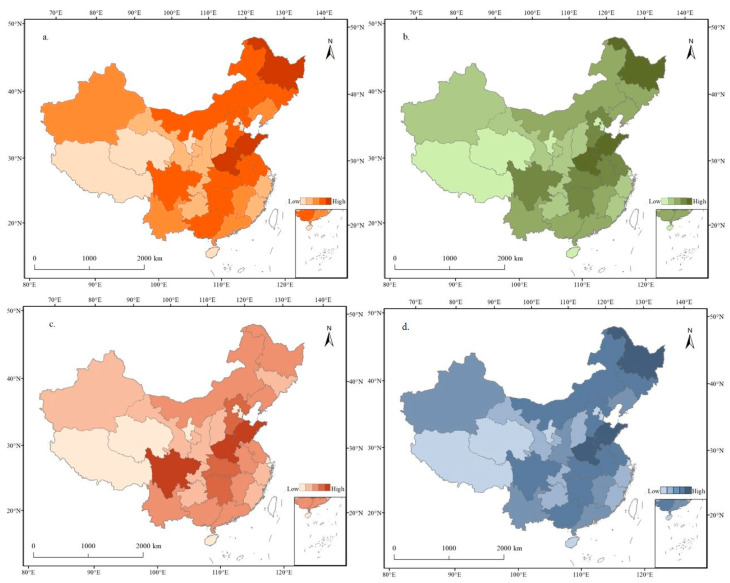
Spatial distribution of food nutrient production in 2020. (**a**) Calorie production, (**b**) protein production, (**c**) fat production, (**d**) carbohydrate production.

The spatial distribution of energy, protein, fat, and carbohydrate supply in China exhibited a consistent pattern, characterized by large differences between the eastern and western regions, with smaller differences observed between the northern and southern regions (Figure 6). Thus, the overall spatial characteristics pattern can be described as “high in the east and low in the west”. In terms of energy supply, the provinces of Henan, Heilongjiang, Shandong, Sichuan, Hebei, and Anhui emerged as the leading contributors, collectively responsible for 43.93% of the total energy production in China. Conversely, Beijing, Shanghai, Tibet, Qinghai, Tianjin, and Hainan had the lowest energy production, accounting for a mere 0.90% of the national total energy production. The fat supply was primarily dominated by Henan, Shandong, Sichuan, Hubei, and Hunan, accounting for 41.74% of the total fat supply in China. In general, the total production of energy and the three major nutrients were mainly concentrated in key regions such as the Northeast Plain, Huanghuaihai Plain, Yangtze Plain, and Sichuan Plain. This concentration of production placed additional pressure on the main marketing areas, such as Beijing and Shanghai, which experienced relatively low supply capacity. Conversely, the Loess Plateau and Qinghai–Tibet Plateau region exhibited low supply capacity and underutilized natural resources.

The spatial distribution of food nutrient production can be attributed to two aspects. On the one hand, the capacity of the food nutrient supply is limited by the natural and economic resources of regional agriculture [48]. The former encompasses elements of the natural environment that can be utilized, such as land, water resources, climate resources, etc.; the latter involves agricultural policies, agricultural labor, capital inputs, agricultural technology, etc. The Northeast Plain, Huanghuaihai Plain, Yangtze Plain, and Sichuan Plain have natural and economic resource advantages. However, China’s agricultural diversification is currently restricted by policies that make it difficult to capitalize on the agricultural advantages of the Loess Plateau and Qinghai–Tibet Plateau regions.

**Figure 7:**
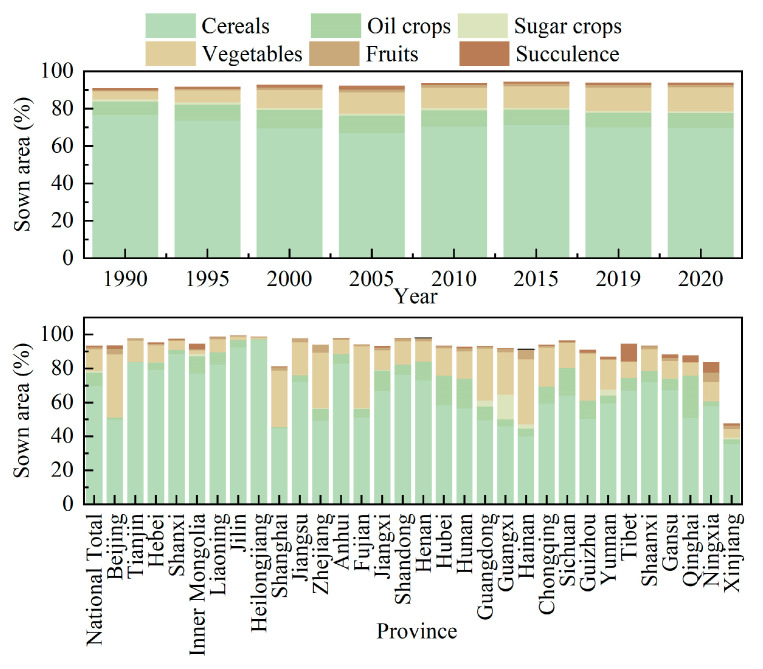
Temporal evolution and spatial distribution in agricultural planting structure.

The agricultural landscape in China is characterized by a predominance of homogeneous agriculture, where cultivation, primarily focused on grain production, is dominated by cereals (Figure 7). However, inconsistencies in the planting structures have led to reduced overall efficiency within the agricultural sector. Meanwhile, at the national level, distinct regional differences exist in agricultural systems, although grain production remains the dominant focus across all regions, followed by the cultivation of vegetables. Heilongjiang and Jilin had the highest proportions of grain cultivation areas, exceeding 90%. Conversely, regions such as Hainan, Beijing, Fujian, Shanghai, and Zhejiang had a significant share of vegetable cultivation, surpassing 30%. Green fodder represents a vital animal feed in China. However, its utilization remained notably low. Tibet, Ningxia, Qinghai, Inner Mongolia, and Gansu, concentrated in the northwestern livestock area of China, possessed a relatively rich resource in green fodder, whereas Hainan, Henan, and Shandong exhibited the lowest levels of green fodder cultivation areas. Additionally, the lowest 23 provinces reported less than 1% green fodder cultivation area. The exclusive emphasis on food production without due consideration of regional resource distribution differences has limited the exploitation of regional advantages, significantly impacting the diversification and comprehensive growth of the agricultural sector and constraining the depth and breadth of agricultural development.

### 3.3. Temporal Evolution in LCC 

**Figure 8:**
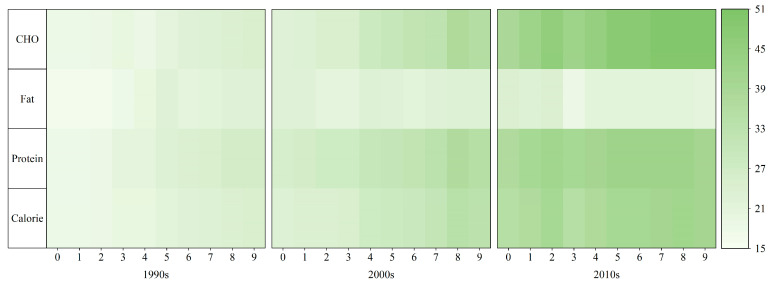
Temporal changes in LCC during the period 1990–2019 (10^3^ million people).

From 1990 to 2020, China experienced considerable changes in land productivity and exhibited a fluctuating upward trend in both energy LCC and LCC changes (Figure 8). Notably, energy LCC and carbohydrate LCC show a declining trend from 2019 and protein shows a declining trend from 2018. During this period, China’s population also increased, although at a relatively slow pace compared to the energy-carrying population. The carbohydrate LCC remained generally in line with the energy LCC; however, owing to agricultural policies and constraints in production structure, the oil crop industry and livestock farming experienced a relatively gradual development, resulting in the successive divergence of fat and protein LCC. Therefore, differences in LCC, based on dietary nutrient structure, can be divided into three phases: a consistent growth phase from 1990 to 2000, a fat-partitioning phase from 2001 to 2013, and a protein-partitioning phase from 2014 to 2020.

During the consistent growth phase, the analysis of energy LCC, in terms of total diet, indicated that it exceeded the actual population and also showed a consistent growth trend, increasing from 1763 million people in 1990 to 2283 million people in 2000, marking a 29.53% increase. This signified that the food production was capable of meeting Chinese nutritional needs, with an increasing surplus. The dietary nutrient structure revealed a uniform growth trend for all three categories during this phase, characterized by steady increases. The protein LCC was notably high, while the fat LCC remained low. In 2000, the LCC for carbohydrates, proteins, and fats amounted to 2268 million, 2571 million, and 2187 million, respectively.

During the fat partitioning phase, it witnessed accelerated changes in energy LCC, increasing from 2358 million people in 2001 to 3517 million people in 2013. The dietary nutritional structure revealed growing disparities among carbohydrate, protein, and fat LCCs. Since 2001, the structural contradiction between food supply and demand has become increasingly prominent, with increasing demand for edible oils and animal foods, whereas agricultural production structure changes remained relatively modest. Consequently, the growth of fat LCC diverged from carbohydrate LCC and protein LCC during this period. The fat LCC became a critical limiting factor in improving the land carrying capacity. This result was consistent with Wang’s findings [49]. In 2013, the carbohydrate LCC and protein LCC increased to 4222 million and 3962 million, respectively, while the fat LCC decreased to 1893 million.

During the protein partitioning phase (2014–2020), the energy LCC displayed slow initial growth, followed by a rapid decline, increasing from 3789 million people in 2014 to 3841 million people in 2020. The dietary nutrient structure showed that the growth rate of carbohydrate LCC during the increasing stage remained essentially consistent with that in the previous stage. However, the growth rate of protein LCC tended to decelerate, and even decrease, leading to further divergence between the two and an intensification of structural contradiction between food supply and demand. In 2020, the carbohydrate LCC increased to 4653 million, whereas the protein and fat LCCs decreased to 3900 million and 2003 million, respectively.

### 3.4. Temporal Evolution and Spatial Differences in LCCI

**Figure 9:**
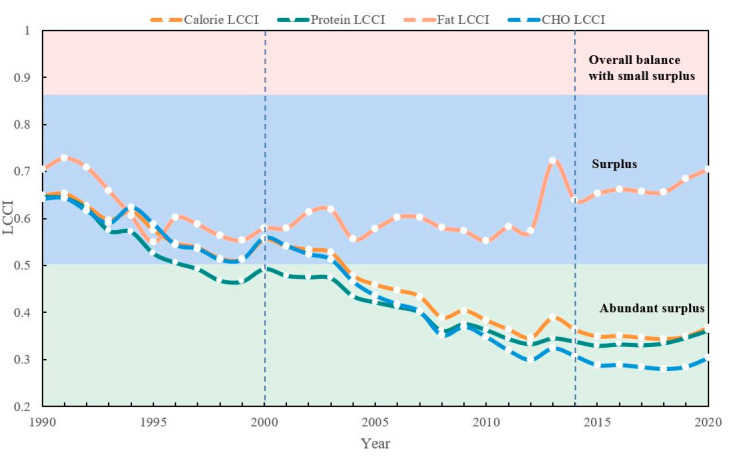
Temporal changes in LCCIs.

The LCC in China maintained a continuous surplus from 1990 to 2020, with an overall trend showing a transition from “decreasing” to “leveling off” (Figure 9). The energy-based LCCI decreased from 0.6487 to 0.3677 during 1990–2013, and it levelled off thereafter. The LCCI trend during 1990–2013 indicated an increasing capacity to satisfy food supply and a decrease in land pressure, shifting from a “surplus” to an “abundant surplus”. This shift was closely associated with the high emphasis placed by the Chinese government on domestic food security. However, after 2014, the increase in LCC encountered limitations, necessitating innovative approaches to further boost LCC. LCCI differences based on dietary nutrition progressively expanded. The protein-based and carbohydrate-based LCCIs reached an ‘abundant surplus’ in 1997 and 2004, respectively. Although differences were observed in the rate of reduction between these two, they eventually converged to the same level after 2014. The fat LCCI was consistently in a “surplus” state, experiencing minimal fluctuations between 1990 and 1999. From 2000 to 2012, it maintained an overall steady level but exhibited more significant fluctuations after 2014. This represented substantial supply pressure variations within the nutritional structure, with a continuing severe shortfall effect due to increased pressure on fat LCC.

**Figure 10:**
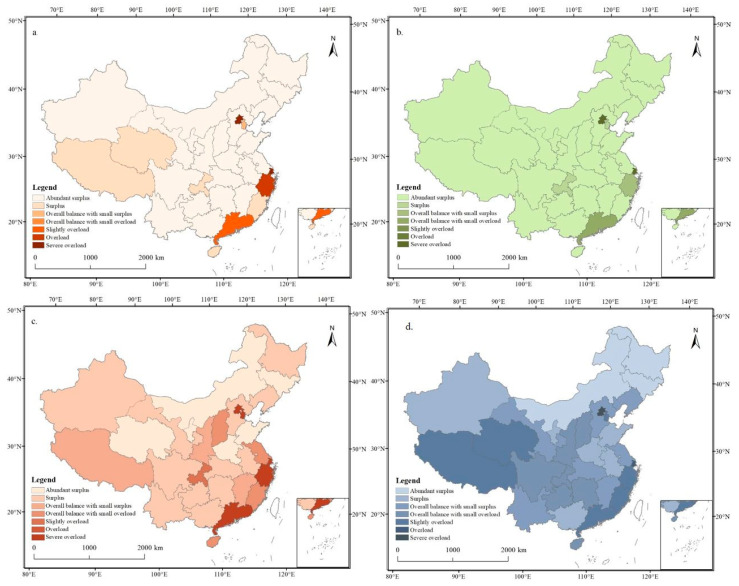
Spatial distribution of LCCIs in 2020. (**a**) Calorie LCCI, (**b**) protein LCCI, (**c**) fat LCCI, and (**d**) carbohydrate LCCI.

The spatial patterns of LCCI for energy, proteins, fats, and carbohydrates were generally consistent, with high LCCI values concentrated in regions including Beijing, Tianjin, Shanghai, Zhejiang, Fujian, Guangdong, Chongqing, and Tibet, indicating a low degree of alignment between food supply and demand in these regions (Figure 10). Conversely, regions with low LCCI values were predominantly situated in the Huanghuaihai Plain and Northeast Plain, encompassing provinces like Henan, Shandong, and Jilin. This signified that these regions presented an excess supply over demand and played a significant role in agricultural production. Specifically, the energy LCCI showed an abundant surplus state in 21 provinces. Notably, Guangdong experienced slight overload, while Zhejiang was in overload; moreover, Beijing and Shanghai were in severe overload. The spatial structures of the protein, carbohydrate, and energy LCCIs were quite similar, while the fat LCCI exhibited substantial disparities. The fat LCCI demonstrated an abundant surplus state in only six provinces of Inner Mongolia, Henan, Jilin, Qinghai, Shandong, and Ningxia, slight overload in Chongqing, and severe overload in the five provinces Beijing, Shanghai, Zhejiang, Tianjin, and Guangdong. This revealed significant disparities in fat supply demand matching across most provinces, pinpointing regions with a pronounced shortfall in regional LCC. 

The spatial patterns of the LCCI converged with those of the food supply, suggesting that food supply contributed more to the LCC based on dietary nutrition. This emphasized the importance of making adjustments at the end of agricultural production to optimize the food system. Furthermore, the LCCI showed a significantly negative correlation with the level of regional economic development and urbanization. This underscored the need to effectively safeguard the overall regional food supply capacity and improve the resilience of urban food security.

## 4. Discussion

### 4.1. Food Supply and Demand Interactions

**Figure 11:**
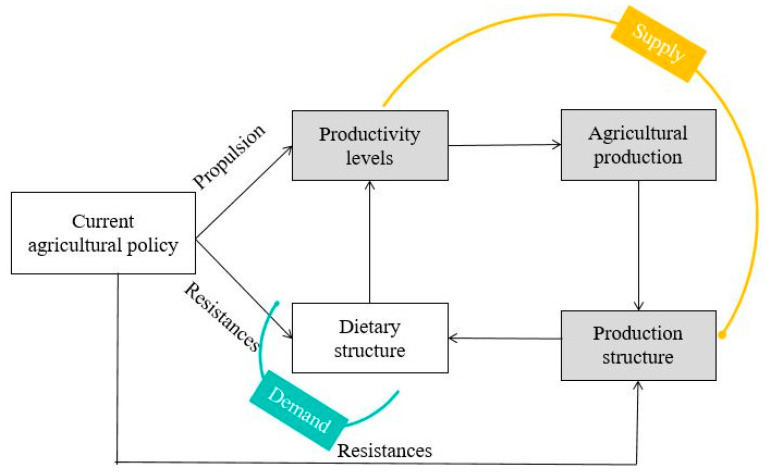
Food supply and demand interactions.

Studying the interaction between food supply and demand holds valuable insights for guiding food system development (Figure 11). On the supply side, increased agricultural productivity led to increased grain production. These surpluses of grain can be converted by farmers to bolster returns and improve efficiency through a combination of agriculture and livestock farming. This transformation in agricultural production reshapes the structure. From an ecological perspective, the dietary habits of animal foods are often reliant on their food sources [50]. Changes in the agricultural products on the supply side catalyze alterations in dietary structures on the consumption side, which, in turn, drive changes in production methods. This dynamic encapsulates market regulation as a natural social law.

On the other hand, national policy support and institutional safeguards play pivotal roles in driving the evolution of the food system [46]. To a certain extent, ensuring food security and adapting the agricultural land structure are land-use behaviors aligned with national macro-strategic objectives. Current agricultural policies that prioritize grain production have become resistant to production restructuring [10]. This limitation means that agricultural products struggle to meet the nutritional and health needs of people, impeding progress in living standards. Technological advancements in agricultural production, propelled by policy support, have been the driving force behind increased productivity levels [51]. At present, China’s agriculture is developing towards the stage of Agriculture 4.0, which is characterized by a high degree of intensification, precision, intelligence, synergy, and ecology, with the support of the internet of things, big data, artificial intelligence, robotics, and other technologies. Agriculture 4.0 can smoothly integrate agricultural resources through networks and information, increase the technical content of resources, and improve the efficiency and quality of agricultural production [52]. However, raising productivity levels within an inappropriate production structure can intensify the irrational use of resources and accelerate the development of food systems in an adverse direction.

China’s existing dietary habits and production exhibit a level of spontaneity and randomness, and there is a lack of scientific guidance and macro-control. At present, the Chinese food system is currently undergoing a critical transformation [53], and there is an urgent need to systematically realign food consumption and production. These changes should be aimed at ensuring that they complement, harmonize, and enhance one another to boost the output rate of resources and the quality of consumption. This approach is essential for achieving optimal benefits from the food system.

### 4.2. Increasing Awareness of the Food System and Guiding Dietary Structure Optimization

The results show that the structure of food consumption in China is still characterized by a “high grain diet”, with carbohydrate consumption higher than the recommended values and fat consumption lower than the recommended values. Dietary imbalance is a major risk factor for the development of chronic diseases [45]. A total of 3.1 million deaths of Chinese residents in 2017 can be attributed to dietary irrationality [45]. In order to enhance food consumption levels, it is important to guide the population towards promoting dietary diversity, optimizing food combinations, and embracing Chinese food development. 

(1) Promote dietary diversity: Encourage a wide variety of foods in your diet, including grains, potatoes, vegetables, fruits, meats, poultry, fish, eggs, dairy, soybeans, and nuts. It is recommended that individuals consume more than 12 different food items per day to ensure a balanced supply of dietary energy and nutrients [31]. In order to promote food diversification, food- and nutrition-related knowledge for Chinese residents can be widely publicized to improve the food consumption behavior of the population. In addition, nutritional improvement programs such as the Soybean Revitalization Program and the Nutritious Meal Program for Students can be implemented to improve the food consumption patterns and nutritional and health status of the population [54].

(2) Optimize food combinations: The nutritional quality of diets may be improved through smart combinations of foods. A key aspect of enhancing the dietary structure in China is increasing the intake of animal products like meat, eggs, and dairy. One should aim for energy ratios of 20–30% from fat and 10–15% from protein, while reducing cereal intake to lower carbohydrate energy ratios of 50–60% [31].

(3) Embrace Chinese food development: Dietary choices may be customized to align with Chinese culinary traditions, prioritizing a plant-based diet but not limiting the intake of animal foods. A balanced grain-to-animal food consumption ratio should be maintained, ideally between 1:0.5 and 1:1, with a focus on high nutritional standards [55]. Food choices may be adapted to local conditions, gradually improving dietary structures in line with regional and population group differences. Notably, the dietary evolution of residents in the middle and lower reaches of the Yangtze River and South China [31] represents a model for Chinese food development with regional characteristics.

### 4.3. Establishment of Large Agricultural Views and Protection of Agricultural Land in All Land Areas and Types 

The primary factor limiting improvements in LCC was fat at the national level. To improve the LCC, the key strategy for transforming both the quantity and structure of agricultural land is to increase the supply of animal food. According to regional LCC differences, the LCCs of the Huanghuaihai Plain and the Northeast Plain were high, whereas South China and the middle and lower reaches of the Yangtze River showed low LCC values. The core of transforming the spatial distribution lies in maximizing the utilization of regional resources, leveraging the self-sufficiency or basic self-sufficiency of agricultural products in each area, and gradually reducing disparities in regional LCC.

(1) Promoting structural transformation of agricultural land quantity. First, we must respect the laws of nature, the economy, and society. In addition, it is crucial to adopt a “relatively crop-neutral” agricultural policy with a foundation in absolute food self-sufficiency [10]. To prevent damage to the tillage layer, we should correct the historical biases that have favored wheat, rice, and maize production, which have hindered the transition to other high-value crops. We should strive for a more equitable playing field for the production of all foodstuffs. This involves reducing the area dedicated to wheat and rice while increasing the cultivation of soybeans, oil crops, and sugar crops. Feed consumption has emerged as a primary factor affecting food security in China, and it is imperative to develop feed production as a significant industry [56]. Given the pressure on feed production, it is essential to separate the planning for grain and feed production. Establishing a ternary planting structure that combines grains, cash crops, and feed crops will promote agriculture and animal husbandry, leading to high yields, enhanced utilization, and palatability of feed, ultimately maximizing livestock and poultry production. Furthermore, we should broaden the scope of agricultural resource utilization to include not only cultivated land but also woodlands, grasslands, and bodies of water [57]. Lastly, we must stabilize pork production and supply through various measures. Pork is a staple in Chinese consumer diets and plays a central role in the meat consumption structure. To ensure stability in pork production, there is a need to strengthen control over pig production capacity, implement monitoring and early warning systems, and provide guidance to farms and households for the implementation of sustainable production, land preservation, environmental protection, and other long-term support policies [58].

(2) To promote the transformation and upgrade of the spatial structure of agricultural land, it is essential to make macro-level adjustments based on natural resource endowments and ecological security. This approach ensures the full realization of the advantages of agricultural production.

- The Northeast Plains region should leverage its cultivated land advantages to focus on grains, soybean, and sugar production, further solidifying its status as China’s primary commercial grain production base. Additionally, considering the relatively limited fat-bearing capacity in the northeast region, there is a need to expand planting areas. This expansion should encompass the production of feed soybeans and feed corn, as well as transitioning some corn monoculture areas into corn–legume–pasture interplanting. These measures will contribute to a stable feed supply and bolster the production of pork, poultry, eggs, and milk.
- In the Huanghuaihai Plain region, there has been a strong emphasis on wheat cultivation and the production of various grains, aimed at enhancing the quality of cultivated land within the region. Moreover, the region has effectively utilized crop stalks and pasture resources to promote the development of grass-fed livestock. Additionally, there have been initiatives to foster shellfish and aquatic breeding in Bohai Bay and the waters of the Shandong Peninsula. Considering large and densely populated cities like Beijing and Tianjin, the establishment of modernized production bases for fresh and live agricultural products, including vegetables and dairy products, has been essential. These endeavors are crucial for ensuring a steady supply of fresh and live agricultural products to meet the demands of urban populations.
- The middle and lower reaches of the Yangtze River benefit from favorable water and heat conditions as well as fertile soils. However, urban expansion has increased pressure on cultivated land protection and reduced the LCC. It is essential to strengthen control over cultivated land use, ensuring that the total cultivated land area remains stable. This will allow the region to continue benefiting from advantages in rice production and maintain the steady growth of the food supply. The region should focus on the development of beans, oilseeds, and potatoes to diversify agricultural production. Additionally, the establishment of intensive and environmentally friendly herbivore production bases on grassy hills and slope areas in the southern part of the region can contribute to sustainable agriculture. To fully utilize its water resources, the region should develop aquatic products, aiming to become a significant supply base for aquatic products in China. Given the high level of urbanization in the middle and lower reaches of the Yangtze River, it is crucial to create intensive urban agricultural security zones on the outskirts of cities. These zones can accommodate vegetable production, large-scale livestock and poultry farms, and standardized aquatic health farms. This approach enhances the region’s food security while addressing the challenges associated with urban expansion [59].
- In South China, the region faces the challenge of a high food demand but relatively low food supply, leading to a low LCC. Thus, agricultural resources in the region must be strictly controlled without reducing the total cultivated land area. It is essential to increase the food self-sufficiency rate, protect cultivated land, and improve the cultivated land replanting index. Leveraging its natural advantages, particularly in the production of fruits like lychee, longan, pineapple, and other tropical fruits, as well as sugarcane, is crucial. Increasing the scale of fruit output can help meet the high food demand. The region should optimize the space for fishery production by rationalizing the areas designated for freshwater and marine aquaculture. Expanding aquaculture in deep-sea and other large water surfaces is essential for enhancing the security of the aquatic product supply.
- In the southwest region, the protein- and fat-carrying capacities are insufficient for achieving a comprehensive LCC. The region should make better use of its climatic resources, pastures, and water bodies to promote the development of grass-fed livestock and freshwater farming. The promotion of corn–soybean strip compound planting can help improve the supply of essential crops. Expanding the forage planting area is crucial to enhance the capacity to produce feed for livestock.
- The Tibetan Plateau and Loess Plateau, despite their vast land areas and abundant resources, currently have low food supply capacities. They face challenges due to lower climatic resource endowments and food production capabilities compared to the national average. However, they possess valuable pasture resources and specialty agricultural products. These regions should focus on harnessing their vast grassland resources to enhance nutritional and biological yields per unit of land area [16]. Building commodity bases centered around grass-fed livestock, such as dairy cows, beef cattle, and sheep, can be beneficial [29]. The presence of numerous wild animals and plants, including mother of pearl, wolfberry, and snow lotus, with economic and medicinal value should be leveraged. Protecting and utilizing these resources for the production of regional specialty agricultural products can meet the diverse food needs of the population.

### 4.4. Innovations and Contributions

The results of this study show that China is experiencing significant shifts in its dietary structure, leading to increased nutritional demands and pressure on the food supply. This coincides with existing research outcomes [17,18,19,31]. This study refines the LCC model found in the literature [60,61,62] by incorporating food nutrition into the evaluation system. One of the findings of this research is that the LCC in China maintained a continuous surplus from 1990 to 2020, but is beginning to have a declining trend. There are differences in the LCCs of carbohydrates, proteins, and fats, with fat being the primary factor limiting the increase in China’s LCC. In addition, our results showed that the LCC varies widely in different provinces and that the restructuring of agricultural production is the key to optimizing the food system. Therefore, specific policy implications, such as leveraging regional advantages and reducing disparities in regional LCCs, are proposed to ensure national food security and the human–food balance.

### 4.5. Limitations and Future Studies

The limitations of this study and future research directions are as follows. (1) The food system is highly complex, influenced by various factors such as climate change, resources, capital, technology, culture, and politics. Our evaluation of the population carrying capacity based on diet and nutrition provides an upper limit under specific conditions. However, it is important to acknowledge that our analysis did not consider inter-regional material circulation and exchange, and regional productivity assumptions remained constant. This leads to a gap between realistic LCC and actual LCC. In future research, we plan to explore LCC under different scenarios. (2) Our study revealed that the natural resource endowment, socioeconomic status, and food production patterns in different regions significantly impact the food consumption and nutrition levels of the population. We intend to conduct further studies to analyze the multiplier effect of LCC based on limiting factors to study the synergistic relationship of trade-offs among socio-economics, food supply, and food demand. (3) While our study accounted for losses in food production related to post-receipt handling, storage, processing, distribution, and consumption, there are various food processing methods. We recognize the need for refining the calculation methods to enhance accuracy. (4) Our data on food consumption were sourced from the China Household Survey Yearbook and the China Statistical Yearbook, which primarily record consumption amounts and do not include quantities consumed while dining out. As living standards improve, out-of-home consumption becomes more prevalent. Thus, future research should consider these factors to minimize research inaccuracies. Additionally, data from 2021 to 2022 were excluded from this study due to data limitations. Moving forward, we will make efforts to ensure the continuity of the sample data over time.

## 5. Conclusions

China’s dietary structure is evolving from a predominantly grain-based diet to an increased consumption of animal products with an accompanying reduction in plant-based food consumption. The proportion of carbohydrates consumed was higher than the maximum recommended ratio of 7.33%, and the proportion of fat was lower than the minimum recommended intake of 6.05%. In order to achieve the recommended energy supply ratio, the carbohydrate consumption should be further reduced and the fat consumption should be increased. Spatially, the nutrient levels were higher in Zhejiang, Shanghai, Guangdong, and Jiangsu, and were lower in Qinghai, Gansu, and Guizhou. Improving fat and protein consumption in less economically developed regions is a current priority.

China has witnessed substantial growth in the overall production of major agricultural products, resulting in increased food supply capacity. However, some areas experienced high growth in fruit, milk, and poultry production, while pork, sugar, and grain production growth remained sluggish. There has been a notable decline in pork supply since 2015. The nutritional supply showed an average annual growth rate of 1.77% for the carbohydrate supply, 2.46% for the protein supply, and 2.80% for the fat supply. However, the food nutritional supply is not in harmony with demand. Spatially, there are significant differences between the east and west of the country, but overall, there is a pattern of “east high and west low”.

The limiting factors for LCC are the carrying capacities of fat and protein. The divergence in fat and protein carrying capacities in 2001 and 2014 makes them the core limiting nutrients for improving regional LCC. By 2020, the energy-carrying population was 3.841 billion, with LCC values of 4.653 billion for carbohydrates, 3.900 billion for protein, and 2.003 billion for fat. The LCCI trends indicate an increase in food supply satisfaction and a reduction in LCC pressure, transitioning from “surplus” to “abundant surplus.” However, since 2014, there has been a bottleneck in the LCC increase, requiring innovative strategies to address it. High-value LCCI areas with food supply–demand asymmetry are concentrated in regions such as Beijing, Tianjin, Shanghai, and Tibet. Low-value LCCI areas, where food supply exceeds demand, are found in regions such as Henan, Shandong, and Jilin. These spatial characteristics of LCCI align closely with the food supply, emphasizing the importance of adjustments in the agricultural sector to optimize food production.

## Figures and Tables

**Table 1:** Food–nutrition conversion parameters for major food categories.

	Plant Foods					Animal Foods					
	Grains	Vegetable Oil	Vegetables	Sugars	Fruits	Pork	Beef and Mutton	Poultry	Eggs	Milk	Aquatic Products
Energy Kcal/100 g	346	899	20	400	53	331	139	145	139	65	103
Protein g/100 g	7.9	0	1.6	0	0.4	15.1	18.5	20.3	13.1	3.3	16.6
Carbohydrate g/100 g	77.2	0	3.4	99.9	13.7	0	1.6	0.9	2.4	4.9	1.6
Fat g/100 g	0.9	99.9	0.2	0	0.2	30.1	6.5	6.7	8.6	3.6	3.3
Edible percentage %	100	100	89	100	85	91	100	100	63	87	100
Food loss percentage %	8.9	12.4	12.7	6.9	12.7	10.9	10.9	10.9	10.9	2.2	2.2
Food waste percentage %	3.2	5.5	10.6	6.0	10.6	7.5	7.5	7.5	7.5	4.9	4.9

**Table 2:** Evaluation criteria and significance of land carrying capacity index (LCCI) grading.

Types	Levels	LCCI	Meaning
Surplus	Abundant surplus	LCCI ≤ 0.500	Plenty of food with some room to grow
	Surplus	0.500 < LCCI ≤ 0.875	
Balance	Overall balance with small surplus	0.875 < LCCI ≤ 1.000	Population–food relationship largely balanced, but with limited development potential
	Overall balance with small overload	1.000 < LCCI ≤ 1.125	
Overload	Slightly overload	1.125 < LCCI ≤ 1.250	Large food deficit and high population overload
	Overload	1.250 < LCCI ≤ 1.500	
	Severe overload	LCCI > 1.500	

## Data Availability

Publicly available sources of the data used in this study are described in the article, for other data used, please contact the corresponding author on reasonable grounds.
